# Acrometastasis in Breast Carcinoma

**DOI:** 10.31486/toj.22.0105

**Published:** 2023

**Authors:** Caroline Galliano, R. Taylor Bragg, Paresh Rangani, Mark Froom

**Affiliations:** ^1^ Louisiana State University School of Medicine, New Orleans, LA; ^2^ Department of Radiology, Louisiana State University Health Sciences Center–New Orleans, New Orleans, LA

**Keywords:** *Breast neoplasms*, *hand bones*, *neoplasm metastasis*

## Abstract

**Background:** Metastasis to the bone in breast cancer patients is common, but metastasis specifically to the appendicular skeleton is rare. A limited number of cases in the literature describe metastatic breast cancer to the distal limbs, also known as acrometastasis. Acrometastasis in a patient with breast cancer should prompt evaluation for diffuse metastatic disease.

**Case Report:** We describe the case of a patient with recurrent triple-negative metastatic breast cancer who presented with thumb pain and swelling. Radiograph of the hand demonstrated focal soft tissue swelling over the first distal phalanx with erosive changes to the bone. Palliative radiation to the thumb resulted in symptom improvement. However, the patient succumbed to widespread metastatic disease. At autopsy, the thumb lesion was confirmed as metastatic breast adenocarcinoma.

**Conclusion:** Metastatic breast carcinoma to the distal appendicular skeleton, specifically to the first digit, is a rare presentation of bony metastasis and can be an indication of late, widespread disease.

## INTRODUCTION

The most common site of metastasis in patients with breast cancer is bone, with up to 75% of patients with stage IV disease developing skeletal metastases and thus having a poor prognosis.^1^ However, metastasis to the distal appendicular skeleton—acrometastasis—is a rare phenomenon. These lesions, which are found distal to the elbow and knee, indicate a rare presentation of disseminated oncologic disease. Few cases of breast cancer metastasis to the thumb have been reported in the literature.^2,3^ We describe the case of a 70-year-old female who presented to our institution with an erosive breast cancer metastasis to the left first distal phalanx.

## CASE REPORT

A 70-year-old African American female with a medical history of diabetes mellitus and recurrent triple-negative metastatic breast cancer presented to the oncology clinic with 2 weeks of left thumb pain. She had been diagnosed with breast adenocarcinoma 5 years prior with a screening mammogram, and a biopsy that confirmed metaplastic carcinoma of her right breast. She underwent a lumpectomy and received adjuvant chemotherapy with docetaxel (Taxotere) and cyclophosphamide (Cytoxan). The patient was lost to follow-up for more than a year before returning to the clinic with a new right breast mass consistent with recurrent disease. After mastectomy and radiation, the patient was again lost to follow-up for 2.5 years.

**Figure 1.**
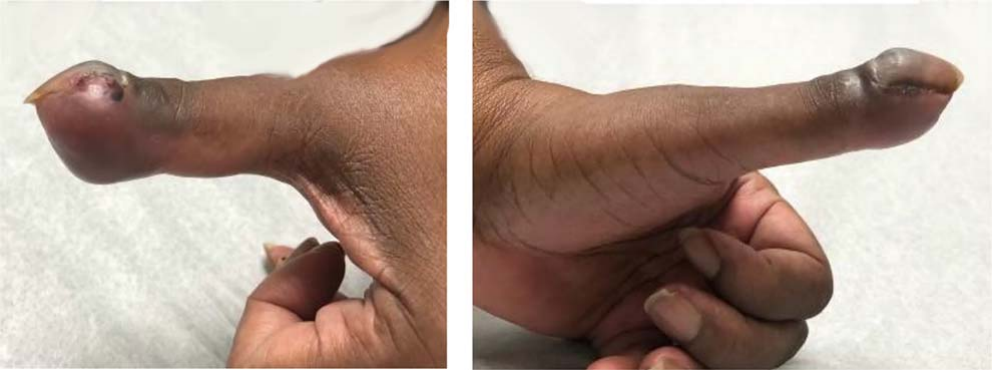
**Gross images of the lesion demonstrate swelling and ulceration at the distal first phalanx**.

**Figure 2.**
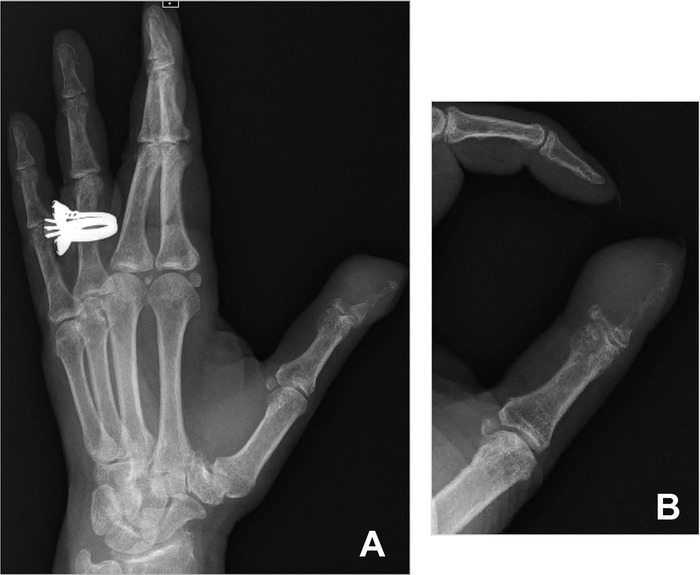
**(A) Oblique and (B) zoomed-in lateral views of the hand and first phalanx demonstrate an eccentric lytic and erosive lesion at the distal first phalanx with surrounding soft tissue edema**.

She subsequently returned to the clinic and presented with complaints of dyspnea on exertion and left thumb pain. Swelling and mild ulceration were noted on physical examination (Figure 1). Left hand radiograph revealed eccentric focal soft tissue swelling and erosive changes involving the first distal phalanx (Figure 2). Given the patient's history of recurrent breast carcinoma and suspicious lung nodules found on subsequent imaging, the phalanx lesion was highly suggestive of osseous metastasis.

After re-establishing care 5 months prior to her presentation with thumb pain, the patient underwent a radionuclide bone scan that showed no sites of abnormal radiotracer uptake suggestive of metastatic bone disease. Following her presentation for thumb pain with radiographic evidence suspicious for metastatic disease, the patient underwent a more aggressive workup with computed tomography (CT) scans of the chest, abdomen, and pelvis, as well as a brain magnetic resonance imaging (MRI) scan. She was found to have metastasis to her lungs, mediastinum, and brain.

The patient underwent 2 rounds of palliative radiation to her left thumb over the course of 2 weeks and 1 round of radiation to her brain. She experienced symptomatic relief in her thumb, but she died approximately 3 months after palliative treatment and 5 years after her initial diagnosis. At autopsy, the patient's lesion to the first phalanx was confirmed to be metastatic breast adenocarcinoma.

## DISCUSSION

Breast cancer is the most common cancer to affect women worldwide, and metastatic disease most commonly involves bone. A large cohort study found that 22% of breast cancer patients developed bone metastasis over a mean follow-up period of 8.4 years.^4^ The median survival for patients with breast cancer and bone metastases is 40 months for patients with triple-negative breast cancer compared to 65 months for patients with estrogen/progesterone-receptor-positive cancer.^5^

Radiographically, osseous metastasis from breast cancer can have several different appearances including osteolytic, osteoblastic, and mixed lesions, with approximately half of the lesions appearing osteolytic.^6^ Radionuclide bone scan is commonly used to detect bone metastases in patients with breast cancer, as the scan is widely available and cost effective. Radionuclide bone scan is the primary technique recommended for women with asymptomatic high-risk breast cancer and typically shows abnormal radiotracer accumulation in sites with increased osteoblastic activity and skeletal vascularity.^7^ Plain x-rays often depict variable findings of metastatic disease, including osteolytic lesions as disrupted or absent trabecular structure or foci of sclerosis.^6^ Detection with CT scan is a more sensitive test (71% to 100%) compared to plain radiographs,^6^ as CT can often detect metastasis in the marrow before bone destruction is apparent. MRI scans can also detect bone marrow metastasis, evidenced by reduced signal on T1, and often with better resolution than CT scans.^6^ Although our patient was lost to follow-up twice, her case demonstrates the paramount importance of radiographic surveillance for metastatic disease throughout the disease course and treatment.

Although osseous breast metastasis is common, metastasis to the distal appendicular skeleton is rare, with metastasis to the hand found in only 0.1% of cases.^8^ The first case of acrometastasis of the hand was reported in 1906.^9^ Since then, only a few other cases have been reported in the literature.^2,3,8,10,11^ Because hand lesions can present similarly to other disease processes with nonspecific symptoms such as pain, swelling, and ulcerations, differentials to this type of bone metastasis include infection, arthritis, gout, and epidermal inclusion cysts. Therefore, diagnosis aided by imaging is crucial because acrometastasis has a poor prognosis and can be associated with widespread disease. Metastatic disease to the distal extremities has been hypothesized to occur through hematogenous spread as opposed to lymphatic spread.^10^ When tumor emboli reach the distal extremities, clinicians should have a high suspicion of tumor deposits elsewhere in the body. Case management is focused on palliative care and thus on symptom control with local radiation, bisphosphonates, curettage, or amputation.^12^

## CONCLUSION

Although bone is the most common site of metastasis in breast cancer, involvement of the distal limb and hand is a rare phenomenon. This finding can be an indication of more widespread disease, prompting additional evaluation for other sites of metastasis. Our case demonstrates a rare occurrence of metastatic breast carcinoma of the first digit and the importance of imaging in a thorough evaluation of this disease presentation.

## Notes

*This article meets the Accreditation Council for Graduate Medical Education and the American Board of Medical Specialties Maintenance of Certification competencies for Patient Care and Medical Knowledge.*
